# Respiratory immune status and microbiome in recovered COVID-19 patients revealed by metatranscriptomic analyses

**DOI:** 10.3389/fcimb.2022.1011672

**Published:** 2022-11-22

**Authors:** Huan Meng, Shuang Wang, Xiaomeng Tang, Jingjing Guo, Xinming Xu, Dagang Wang, Fangfang Jin, Mei Zheng, Shangqi Yin, Chaonan He, Ying Han, Jin Chen, Jinyu Han, Chaobo Ren, Yantao Gao, Huifang Liu, Yajie Wang, Ronghua Jin

**Affiliations:** ^1^ Department of Clinical Laboratory, Beijing Ditan Hospital, Capital Medical University, Beijing, China; ^2^ Beijing Ditan Hospital, Capital Medical University, Beijing, China; ^3^ Translational R&D Center, Guangzhou Vision Medicals Co. LTD, Guangzhou, China

**Keywords:** coronavirus disease 2019, recovery, metatranscriptome, immune status, microecology

## Abstract

Coronavirus disease 2019 (COVID-19) is currently a severe threat to global public health, and the immune response to COVID-19 infection has been widely investigated. However, the immune status and microecological changes in the respiratory systems of patients with COVID-19 after recovery have rarely been considered. We selected 72 patients with severe COVID-19 infection, 57 recovered from COVID-19 infection, and 65 with non-COVID-19 pneumonia, for metatranscriptomic sequencing and bioinformatics analysis. Accordingly, the differentially expressed genes between the infected and other groups were enriched in the chemokine signaling pathway, NOD-like receptor signaling pathway, phagosome, TNF signaling pathway, NF-kappa B signaling pathway, Toll-like receptor signaling pathway, and C-type lectin receptor signaling pathway. We speculate that *IL17RD*, CD74, and TNFSF15 may serve as disease biomarkers in COVID-19. Additionally, principal coordinate analysis revealed significant differences between groups. In particular, frequent co-infections with the genera *Streptococcus*, *Veillonella*, *Gemella*, and *Neisseria*, among others, were found in COVID-19 patients. Moreover, the random forest prediction model with differential genes showed a mean area under the curve (AUC) of 0.77, and *KCNK12*, *IL17RD*, *LOC100507412*, *PTPRT*, *MYO15A*, *MPDZ*, *FLRT2*, *SPEG*, *SERPINB3*, and *KNDC1* were identified as the most important genes distinguishing the infected group from the recovered group. *Agrobacterium tumefaciens, Klebsiella michiganensis, Acinetobacter pittii, Bacillus* sp. FJAT.14266, *Brevundimonas naejangsanensis, Pseudopropionibacterium propionicum, Priestia megaterium, Dialister pneumosintes, Veillonella rodentium*, and *Pseudomonas protegens* were selected as candidate microbial markers for monitoring the recovery of COVID patients. These results will facilitate the diagnosis, treatment, and prognosis of COVID patients recovering from severe illness.

## Introduction

Severe acute respiratory syndrome coronavirus 2 (SARS-CoV-2), a highly contagious respiratory pathogen, is a beta-corona virus that causes coronavirus disease 2019 (COVID-19). Previous clinical experience has shown that COVID-19 is highly heterogeneous in symptomatic cases, ranging from mild to severe to lethal (Zhou et al., 2020). Infection with certain respiratory viruses often produces a strong inflammatory response (Huang et al., 2005; de Jong et al., 2006). Deficiencies in immune regulation, known as hypercytokinemia or “cytokine storms,” are often associated with deleterious outcomes, such as acute respiratory distress syndrome (ARDS) (de Jong et al., 2006). Most viruses, especially SARS, disrupt the normal immune response of the host, including interferon (IFN) signaling (Shaw et al., 2017; Crosse et al., 2018). The IFN system affects viral replication and downstream antiviral immunity (Costa-Pereira et al., 2002; Grandvaux et al., 2002). The type I interferon (IFN-I) response mainly exerts an antiviral function by inducing the expression of interferon-stimulated genes (ISG), which plays an important role in fighting viral infection (Samuel, 2001). Viral infection and replication lead to differences in the dynamics and magnitude of host responses, which affect the outcome of innate and adaptive immune responses. For example, SARS coronavirus effectively inhibits IFN inducibility and antagonizes IFN effects by utilizing its structural and nonstructural proteins (de Wit et al., 2016). SARS-CoV is unable to elicit strong induction of an IFN response in human macrophages (Cheung et al., 2005; Ziegler et al., 2005) and in SARS patients (Reghunathan et al., 2005). Insufficient IFN responses may lead to progressive increases in viral load with hypercytokinemia and ultimately fatal consequences in patients with SARS. Clinical studies on COVID-19 suggest the presence of severe cytokine MIa (Huang et al., 2020). To date, the mechanisms by which pathogen infection disrupts host immune homeostasis and stimulates excessive inflammation-induced responses remain unknown.

Infectious diseases other than COVID-19 have been identified as post-infection syndromes (Bannister, 1988; Hickie et al., 2006). In a study of COVID-19, Q fever, glandular fever, epidemic polyarthritis, and Legionella were similar to the persistent symptoms of COVID-19 (Hickie et al., 2006; Loenhout et al., 2014). Repeated symptoms include burnout, pain, limited quality of life, and limited health. Therefore, COVID-19 may be a persistent symptom. Additionally, loss of taste and/or smell has not been described in studies of post-infectious syndromes of diseases other than COVID-19; therefore, it is likely to be a persistent symptom of COVID-19. Some patients, even those with COVID-19, still exhibit symptoms after their initial recovery (Greenhalgh et al., 2020; Tenforde et al., 2020). Post-infection syndromes have been identified in many other infectious diseases (Bannister, 1988; Hickie et al., 2006). For example, after a mild SARS-CoV infection, a significant proportion of patients experience residual lung damage (Chan et al., 2003; Zhang et al., 2020). Additionally, some studies have shown that patients infected with SARS-CoV experience psychological symptoms, burnout, health limitations, and a decreased quality of life (Lau et al., 2005; Lee et al., 2007; Lam et al., 2009). Prospective studies have shown burnout, neurocognitive impairment, musculoskeletal pain, and mood disturbances in patients six months after infection. These conditions cannot be extrapolated to patients with COVID-19 (Hickie et al., 2006), but a proportion of patients with COVID-19 are expected to experience physical, cognitive, or psychological distress within three weeks after recovery. However, no studies have described the changes in the immune status of the respiratory tract or changes in the respiratory tract microecology from the perspective of molecular mechanisms in patients with COVID-19 after recovery.

Metatranscriptomic sequencing provides an agnostic approach to detect pathogens that emerge directly from clinical samples (Wilson et al., 2014). Compared with targeted approaches, it also provides valuable information on microbiota composition and may reveal superinfections that may affect disease development and prognosis (Westermann et al., 2012; Avraham et al., 2016). One of the key advantages of metatranscriptomic sequencing is that a snapshot of the patient’s microbiome can be obtained at a specific sampling point to detect superinfections and identify other microorganisms that may affect patient outcomes. In addition to evidence of true pathogens, there is growing evidence that the microbiota of the respiratory tract may affect patient health; however, most of the evidence focuses on interactions between bacteria (Kumpitsch et al., 2019). Increasing evidence suggests that analyzing the microbiome makes it possible to predict which patients with respiratory infections are more likely to develop the severe disease (Kumpitsch et al., 2019; Man et al., 2019). Bacterial load and lung microbiota composition may influence the likelihood of developing acute respiratory distress syndrome (ARDS) in patients with severe artificial respiration (Panzer et al., 2018; Dickson et al., 2020). Metatranscriptomic analysis of COVID-19 offers an opportunity to assess whether the microbiome is beneficial or detrimental to patient outcomes (Li et al., 2019).

In our study, we applied metatranscriptomic sequencing in 194 patients (72 in an infectious period of COVID-19, 57 in a recovery period following COVID-19, and 65 with non-COVID-19 pneumonia) to obtain unbiased, cross-organism transcriptional profiles. We aimed to identify the three core elements of the causative agent of COVID-19–pathogen, respiratory microbiota, and host response–and to apply this information to advance the understanding and diagnosis of COVID-19. This study describes the changes in the immune status and microecology of the respiratory tract after the recovery of patients from the perspective of molecular mechanisms. Also, this study is expected to improve the understanding of the molecular mechanism of the new crown infection process and provide genes and clues for subsequent treatment.

## Materials and methods

### Participants

A total of 171 patients, balanced for age and sex, were enrolled in this study, including those who were infected with COVID-19 or other pathogens, including human coronavirus, human herpesvirus, rhinovirus, parainfluenza virus, respiratory syncytial virus, *Acinetobacter baumannii*, *Staphylococcus aureus*, and *Klebsiella pneumoniae*, or who were recovering from COVID-19. The samples collected from patients with confirmed COVID-19 and convalescent patients who were recovering from COVID-19 infection were classified into the infected and recovered groups, respectively. The samples from patients infected with other pathogens were classified as the other group. All patients enrolled in the study provided written informed consent approved by the institutional review board of the Beijing Ditan Hospital Capital Medical University.

For the infected group, 65 Consecutive patients older than 18 years with community acquired pneumonia or hospital acquired pneumonia were enrolled. All enrolled patients met the study inclusion and exclusion criteria. Inclusion criteria of patients with community acquired pneumonia: (a) community onset; (b) a new infiltrate, lobar or segment consolidation, ground glass opacity or interstitial images revealed on chest radiograph or CT scan, accompanied by pleural effusion or not; (c) any of the following 4 clinical features: (c1) new occurrence of cough, expectation, or worsen of respiratory symptoms, accompanied by purlurent sputum, chest pain, dyspnea and hemoptysis or not; (c2) fever; (c3) signs of consolidation and/or moist rale on lung auscultation; (c4) peripheral white cell counts > 10 × 10ˆ9/L or < 4 × 10ˆ9/L. Inclusion criteria of patients with hospital acquired pneumonia:(a) a new or progressive infiltrate, consolidation, or ground glass opacity revealed on chest radiograph or CT scan; (b) two or more of the following 3 criteria: (b1) fever > 38°C; (b2) purulent airway secretions; (b3) peripheral white blood cell count > 10 × 10ˆ9/L or < 4 × 10ˆ9/L; (c) illness occurs 48 hours or more after admission during hospitalization. Exclusion criteria: (a) patients infected with HIV; (b) pregnant women; (c) patients with an irreversible contraindication for bronchoscopy; (d) patients who were unable to understand the informed consent description or unwilling to sign the informed consent form; (e) patients ultimately diagnosed with a non-infectious disease other than pneumonia.

For the other group, individual who met for the following inclusion criteria and exclusion criteria were enrolled. Inclusion criteria: (a) patients with clinical features such as fever, cough, expectation, pharyngalgia, or diarrhea; (b) peripheral white blood cell count > 10 × 10ˆ9/L or < 4 × 10ˆ9/L. Exclusion criteria: patients infected with COVID-19 as described above.

Nasopharyngeal (NP) swabs or sputum samples were collected from the participants. NP swabs were collected using a sterile swab. The participants were requested to rinse their mouths with water two or three times and expectorate sputum into a sterilized cup. All samples were immediately sent for RNA extraction or stored at -70°C. A total of 194 samples were obtained, including 72, 57, and 65 samples from the infected, recovered, and other groups, respectively.

### Metatranscriptomic sequencing

NP swabs or sputum samples were inactivated using AVL buffer containing guanidinium salts (Vision Medicals, China) and then subjected to RNA extraction (Vision Medicals, China). Library preparation was performed using a Novel coronavirus (2019-nCoV) detection kit (Vision Medicals, China) with 300 μL of the samples, and then 50 bp single-end sequencing was performed with Illumina HiSeq 2500 or MGISEQ-200. Briefly, the extracted RNA was treated with DNase I to remove the contaminating DNA. The mRNAs were purified, fragmented, and rRNA-depleted. RNA was reverse-transcribed and enriched by PCR to construct the final cDNA libraries.

### Host transcriptome analysis

Raw sequencing reads were processed using fastp (https://github.com/OpenGene/fastp). Novel coronavirus (2019-nCoV) nucleic acid analysis software (Vision Medicals, China, v1.0) was used to extract the reads from the host, which were then aligned to the human genome (GRCh38) using HISAT2 v2.1.0. Gene quantification was performed using featureCounts (v2.0.0). DEseq2 v1.32.0 was used to identify significantly differentially expressed genes (|logFC| > 2 and padj < 0.05), which were further subjected to Gene Ontology (GO) (http://www.geneontology.org/) and KEGG (http://www.genome.jp/kegg/) enrichment analyses using clusterProfiler v4.0.5. The expression patterns of 255 immune-related genes from ImmPortDB and 380 interferon-stimulated genes (ISG) (X Wu, 2018 Intrinsic Immunity Shapes Viral Resistance of Stem Cells S0092-8674(17)31365-X) were compared across groups.

### Microbiome analysis

After removing the reads from the host, the clean reads were aligned against reference databases, including plasmids, bacteria, fungi, parasites, and viruses. Alpha diversity indices, including richness, ACE, Chao1, Shannon, and Simpson, were calculated for each sample using Vegan (v2.5-7). Principal coordinates analysis (PCoA) was performed using OTU abundances based on the distance of the Jensen-Shannon divergence (JSD). Linear discriminant analysis effect size (LEfSe, v1.0.8) was applied to identify the discriminant bacterial taxa for different sample groups.

### Classifier analysis

The random forest model was used to predict patient status, including the infected, recovered, or other groups. The models were trained based on differentially expressed genes, differential microbial taxa, or both, resulting in three kinds of classifiers. The random forest classifier was implemented using Python (version 3.7, Python Software Foundation, https://www.python.org/) with the scikit-learn package (https://github.com/scikit-learn/scikit-learn). Feature (gene or taxa) selection was performed using the SelectFromModel method, and features with low importance (according to mean decrease in accuracy (MDA)) were removed before model construction. The area under the curve (AUC) was calculated as an indicator of model performance. We repeated the experiment 100 times for each type of classifier and measured the mean AUC and standard error of the mean.

### Statistical analysis

The Wilcoxon test was used to identify differential genera and species between different groups, and a p-value < 0.05 was considered statistically significant. The Wilcoxon test was also used to compare alpha diversity between groups.

## Results

### Overview of sequencing data

The average number of total reads was approximately 27.3 million for all the samples (**Table S1**). The average mapped ratios were 72.13%, 88.11%, and 80.96% of the total reads for the infected, recovered, and other groups, respectively (**Table S1**). The average proportions of reads mapped to the host were 18.05%, 10.21%, and 50.29% for the infected, recovered, and other groups, respectively (**Table S1**). The average proportion of reads mapped to plasmids and bacteria was 54.05%, 37.71%, and 70.74% for the infected, recovered, and other groups, respectively, while those mapped to viruses were approximately 0.02%, 0.07%, and <0.01%, respectively (**Table S1**).

### Signatures of host immune response

**Figure 1:**
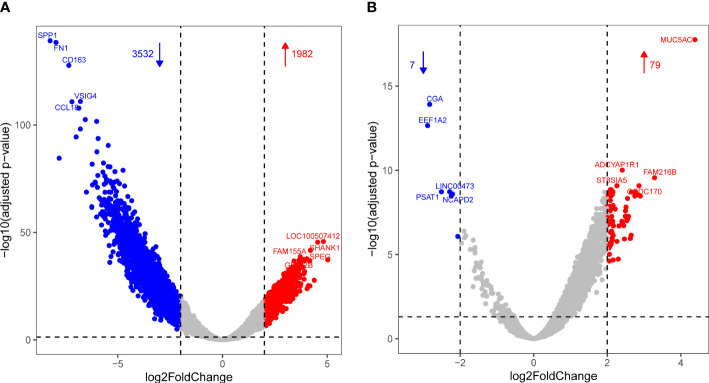
Volcano plot showing differential expression with increased DEGs colored in red and decreased DEGs colored in blue for comparisons of infected vs. other **(A)** and infected vs. recovered **(B)** groups, respectively.

Differential gene expression analysis was performed between the combinations of infected, recovered, and other groups. A total of 1,982 upregulated and 3,532 downregulated genes were detected between the infected and other groups (**Figure 1A**), and 79 upregulated and seven downregulated genes were detected between the infected and recovered groups (**Figure 1B**).

**Figure 2:**
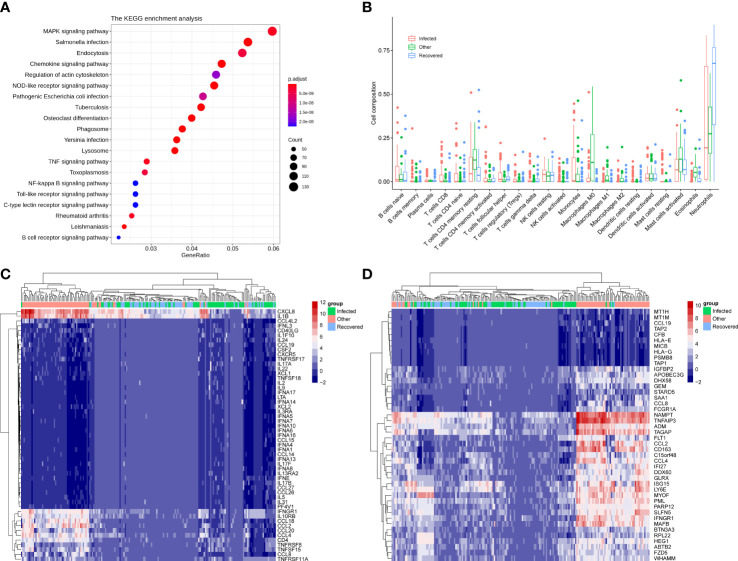
Immune signatures associated with differentially expressed genes. **(A)** KEGG enrichment analysis of the differential expressed genes between infected and other groups. **(B)** Cell composition was estimated by the CIBERSORT algorithm of samples for infected, other, and recovered groups. **(C)** Heatmap showing the expression pattern of 53 differential immune genes across groups. **(D)** Heatmap showing the expression pattern of 44 differential interferon-stimulated genes (ISG) across groups.

The differentially expressed genes between infected and other groups were enriched in the chemokine signaling pathway, NOD-like receptor signaling pathway, phagosome, TNF signaling pathway, NF-kappa B signaling pathway, Toll-like receptor signaling pathway, and C-type lectin receptor signaling pathway (**Figure 2A**). However, analysis of the KEGG pathways associated with the differentially expressed genes between the infected and recovered groups did not show any significant enrichment. Interestingly, some upregulated genes in the infected group compared with recovered group were involved in immune-related functions, including *IL17RD* (interleukin 17 receptor D), *CD74* (CD74 molecule), *NTRK2* (neurotrophic receptor tyrosine kinase 2), *NKD1* (NKD inhibitor of WNT signaling pathway 1), *CD180* (CD180 molecule), *TNFSF15* (TNF superfamily member 15), *HLA-DQA1* (major histocompatibility complex, class II, DQ alpha 1), *HLA-DRB1* (major histocompatibility complex, class II, DR beta 1), *CXCL5* (C-X-C motif chemokine ligand 5), *PIGR* (polymeric immunoglobulin receptor), *ILDR2* (immunoglobulin like domain containing receptor 2), *IGSF1* (immunoglobulin superfamily member 1), *ISLR* (immunoglobulin superfamily containing leucine rich repeat), *IGFN1* (immunoglobulin like and fibronectin type III domain containing 1), *ISLR2* (immunoglobulin superfamily containing leucine rich repeat 2), *IGDCC4* (immunoglobulin superfamily DCC subclass member 4), *IGDCC3* (immunoglobulin superfamily DCC subclass member 3), *IGSF9* (immunoglobulin superfamily member 9), and *IGSF22* (immunoglobulin superfamily member 22).

Immune cell abundance was further estimated using the CIBERSORT algorithm, which revealed that neutrophils were the most abundant immune cell subset across groups and showed significant differences for comparisons of the infected vs. recovered and other vs. recovered groups (**Figure 2B** and **Supplementary Figure 1A**, *p-value < 0.001*). The abundance of M0, M1, and M2 macrophages in the other group was significantly higher than that in the infected and recovered groups (**Figure 2B** and **Supplementary Figures B, C**, *p-value < 0.01*). In addition, 53 immune-related genes (**Figure 2C**) and 44 interferon-stimulated genes (ISG) (**Figure 2D**) were significantly differentially expressed between the combinations of infected, recovered, and other groups (*p-value < 0.001*). The above results indicate that patients with COVID-19 infection and recovered COVID-19 patients showed a distinct host immune response compared to other infections.

### Changes in the microbiome associated with COVID-19

**Figure 3:**
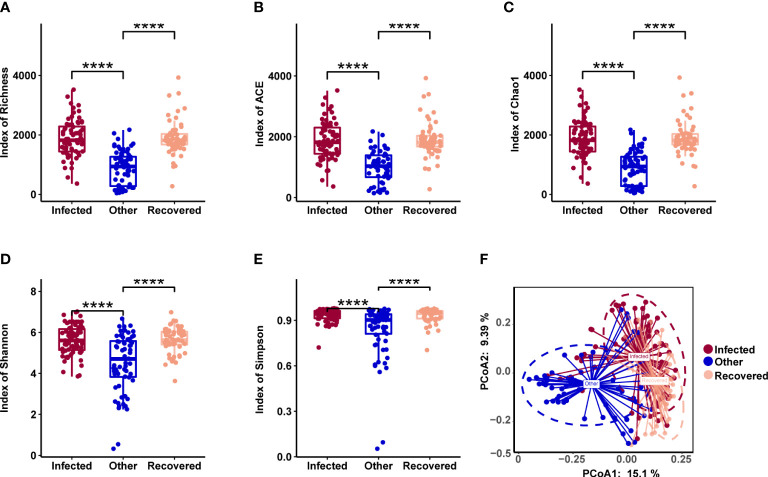
Microbial diversity associated with COVID-19. **(A-E)** Alpha diversity includes richness **(A)**, ACE **(B)**, Chao1 **(C)**, Shannon **(D)**, and Simpson **(E)**. **** indicates a *p-value* < 0.0001 (Wilcoxon test). **(F)** Principal coordinates analysis (PCoA) of samples using a Jensen-Shannon Divergence (JSD) distance.

The microbiome composition in the respiratory tract was evaluated in the three groups. All types of alpha diversity indexes, including richness, ACE, Chao1, Shannon, and Simpson, showed no significant differences between the infected and recovered groups, while those of both these groups showed significant differences when compared with the other group (**Figures 3A-E**, Wilcoxon test, *p-value* < 0.05). PCoA revealed significant differences between all three groups, especially distinguishing the other group (**Figure 3F**).

**Figure 4:**
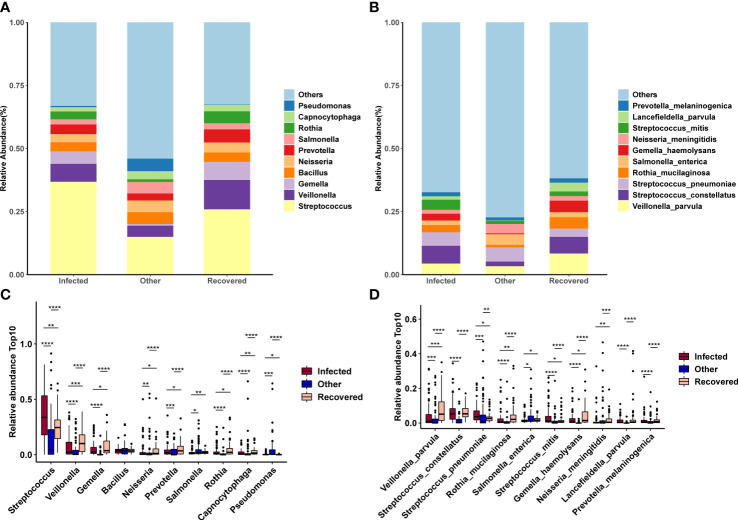
Changes in microbial composition across groups. **(A, B)** Microbial composition of the top 10 taxa at the genus **(A)** and species **(B)** levels. **(C, D)** Differences in the microbial composition of the top 10 taxa between groups at the genus **(C)** and species **(D)** levels. *, **, ***, and **** indicates a *p-value* < 0.05, 0.01, 0.001, and 0.0001 (Wilcoxon test), respectively.

At the genus level, Streptococcus, Veillonella, Gemella, Bacillus, and Neisseria showed the highest abundances (**Figure 4A**). At the species level, Veillonella parvula, Streptococcus constellatus, Streptococcus pneumoniae, Rothia mucilaginosa, Salmonella enterica, Streptococcus mitis, Gemella haemolysans, Neisseria meningitidis, Lancefieldella parvula, and Prevotella melaninogenica were the most abundant (**Figure 4B**). However, most of the top 10 abundant genera (**Figure 4C**) and species (**Figure 4D**) were observed to be significantly different between all combinations of the above three groups (Wilcoxon test, p-value < 0.05).

**Figure 5:**
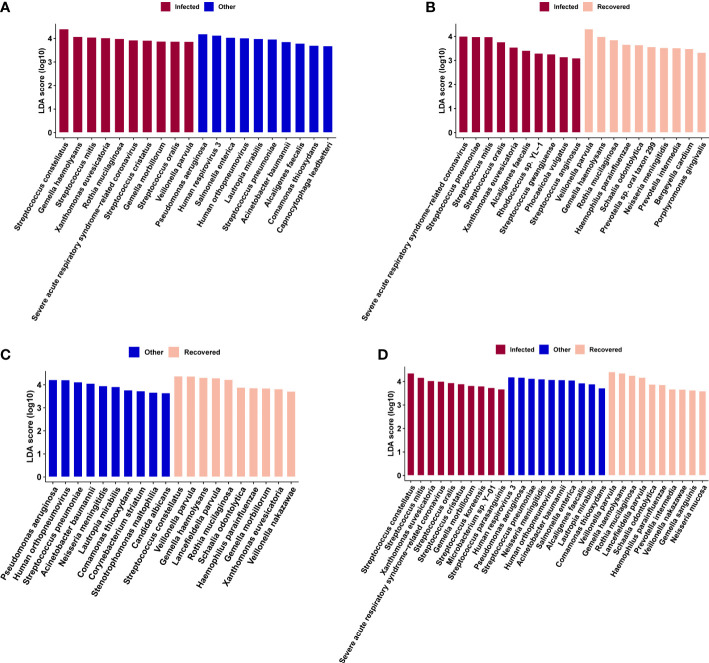
Microbial biomarkers identified by linear discriminate analysis effect size (LEfSe) analysis for combinations of infected vs. other **(A)**, infected vs. recovered **(B)**, and other vs. recovered **(C)** groups, and across the above three groups **(D)**, respectively.

Linear discriminant analysis effect size (LEfSe) analysis was performed to further identify microbial taxa as biomarkers for different groups (**Figure 5**). Specifically, *Human respirovirus 3*, *Pseudomonas aeruginosa*, *Streptococcus pneumoniae*, *Neisseria meningitidis*, *Human orthopneumovirus, Acinetobacter baumannii*, *Salmonella enterica*, *Alcaligenes faecalis, Lautropia mirabilis*, and *Comamonas thiooxydans* were enriched in the other group (**Figure 5D**). *Streptococcus constellatus*, *Streptococcus mitis*, *Xanthomonas euvesicatoria*, severe acute respiratory syndrome-related coronavirus, *Streptococcus oralis*, *Streptococcus cristatus*, *Gemella morbillorum*, *Streptococcus koreensis*, *Microbacterium* sp. Y−01, and *Streptococcus parasanguinis* were enriched in the infected group (**Figure 5D**). In addition, *Veillonella parvula*, *Gemella haemolysans*, *Rothia mucilaginosa*, *Lancefieldella parvula*, *Schaalia odontolytica*, *Haemophilus parainfluenzae*, *Prevotella intermedia*, *Veillonella nakazawae*, *Gemella sanguinis*, and *Neisseria mucosa* were enriched in the recovered group (**Figure 5D**).

### Host transcriptional and microbial classifier associated with COVID-19 recovering

**Figure 6:**
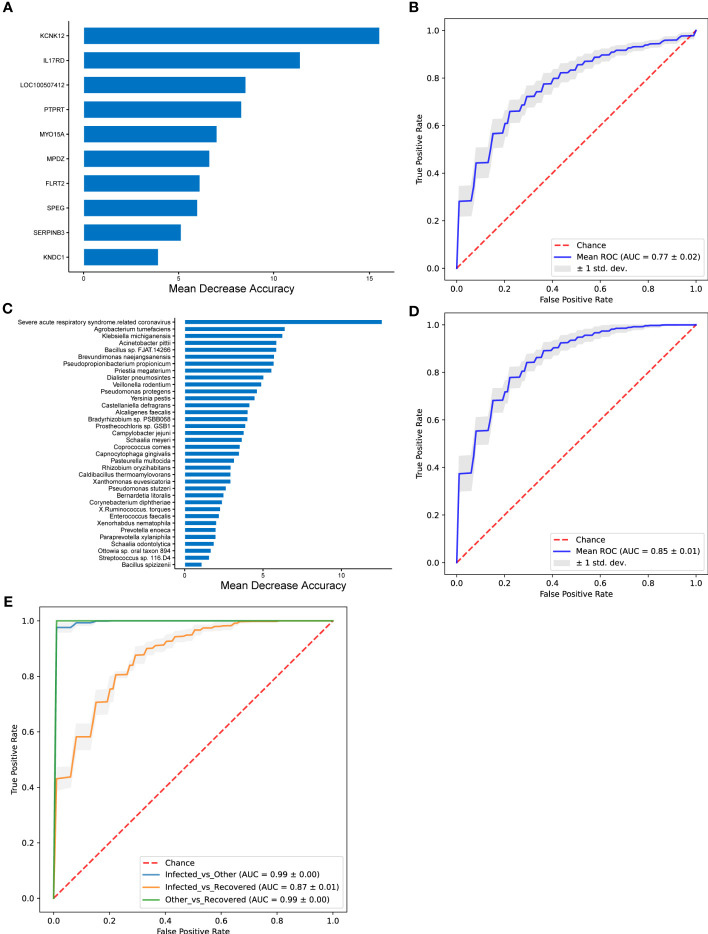
The performance of random forest classifier and associated important features. **(A, B)** Performance of random forest model with differential genes for infected vs. recovered groups. **(C, D)** Performance of random forest model with differential microbial taxa for infected vs. recovered groups. **(E)** Performance of random forest model with both differential genes and differential microbial taxa. The important features were selected using MDA (mean decrease in accuracy) algorithm **(A, C)**, and mean ROC curves are shown with ± 1 standard deviation in gray shading **(B, D)**. AUC, area under the curve; ROC, receiver operating characteristic; std. dev., standard deviation.

Differential genes, microbial taxa, and combinations of differential genes and microbial taxa were used to train the random forest classifier (**Figure 6**). The MDA algorithm was performed to select the more reliable features. The random forest prediction model with the differential genes showed a mean AUC of 0.77 (**Figure 6B**). *KCNK12* (potassium two pore domain channel subfamily K member 12), *IL17RD*, *LOC100507412*, *PTPRT* (protein tyrosine phosphatase receptor type T), *MYO15A* (myosin XVA), *MPDZ* (multiple PDZ domain crumbs cell polarity complex component), *FLRT2* (fibronectin leucine-rich transmembrane protein 2), *SPEG* (striated muscle enriched protein kinase), *SERPINB3* (serpin family B member 3), and *KNDC1* (kinase non-catalytic C-lobe domain containing 1) were identified as the most important genes distinguishing the infected group from the recovered group (**Figure 6A**). The model with the differential microbial taxa showed a mean AUC of 0.85 (**Figure 6D**) and severe acute respiratory syndrome-related coronavirus, *Agrobacterium tumefaciens, Klebsiella michiganensis, Acinetobacter pittii, Bacillus* sp. FJAT.14266, *Brevundimonas naejangsanensis, Pseudopropionibacterium propionicum, Priestia megaterium, Dialister pneumosintes, Veillonella rodentium*, and *Pseudomonas protegens* were identified as the most important taxa that distinguished the infected group from the recovered group (**Figure 6C**).

A random forest prediction model with both the differential genes and microbial taxa exhibited better performance than those with differential genes or taxa independently, which achieved AUC of 0.99 ± 0.00, 0.87 ± 0.01, and 0.99 ± 0.00 for comparisons of infected vs. other, infected vs. recovered, and other vs. recovered groups, respectively (**Figure 6D** and **Supplementary Figure 2**).

## Discussion

In the past two years, COVID-19 has aggressively spread to most countries worldwide and was declared a pandemic by the WHO in March 2020 (WHO, 2020). SARS-CoV-2 is highly pathogenic and infectious, and the clinical features of COVID-19 differ from those of SARS, Middle East respiratory syndrome (MERS), and seasonal influenza (Huang et al., 2020). The metatranscriptomic characteristics of patients with COVID-19 were reported, and COVID-19 patients have a higher potential for concurrent infection and specific triggers of host immune responses through specific pathways compared with non-COVID-19 pneumonia patients (Zhang et al., 2020). Previous studies have implicated the NF-κB pathway in the etiology of severe COVID-19 phenotypes (Hirano and Murakami, 2020). The NF-κB pathway inhibition has a potential therapeutic role in alleviating severe COVID-19 disease (Hariharan et al., 2021). siRNA depletion of retinoic acid-induced I-like receptor (RLR) or aptamers significantly attenuates cytokine and/or chemokine induction, and RLR signaling also contributes to MERS-CoV-induced inflammation-evoked responses (Zhao et al., 2020). IL1B, P2RX7, IFNB1, IFNB1, TNF, and CASP1 enhance the network connectivity between multiple sclerosis (MS) complex genomes associated with COVID-19 and NOD-like receptor (NLR) signaling. The activity of NLR signaling has been found in COVID-19 and MS, revealing that the NLR signaling pathway plays an important role in COVID-19 disease-associated multiple sclerosis (Qiu et al., 2022). Toll-like receptor (TLRs) pathways, as components of innate immunity, could be involved in the pathogenesis of SARS-CoV-2, and several studies have shown that TLRs play an important role in the pathogenesis of SARS-CoV and MERS-CoV (Khanmohammadi and Rezaei, 2021). Conti et al. found that activation of TLRs during COVID-19 infection may produce inflammation-inducing cytokines such as IL-1β (Conti et al., 2020). Additionally, the immunopathological consequences that lead to death in COVID-19 patients result from interactions between TLRs and virions (Patra et al., 2020). In our study, differentially expressed genes between infected and other groups were enriched in the chemokine signaling pathway, NOD-like receptor signaling pathway, phagosome, TNF signaling pathway, NF-kappa B (NF-κB) signaling pathway, Toll-like receptor signaling pathway, and C-type lectin receptor signaling pathway (**Figure 2A**).

Based on a previous study, the rate of bacterial co-infection among hospitalized COVID-19 patients has been estimated to be approximately 7% (Lansbury et al., 2020). More seriously, the proportion of patients with bacterial co-infection increases to 14% in the intensive care unit (ICU) (Lansbury et al., 2020). To examine possible concurrent infections in COVID-19 patients, the microbiota community structure at the genus and species levels was analyzed. Our current data reveal frequent co-infection with *Streptococcus* (*Streptococcus constellatus, Streptococcus pneumoniae*, and *Streptococcus mitis*), *Veillonella* (*Veillonella parvula*), *Gemella* (*Gemella haemolysans*) and *Neisseria* (*Neisseria meningitidis*). This result is consistent with those of previous studies (Desai et al., 2020; Brueggemann et al., 2021; Ma et al., 2021). It is strongly believed that *Streptococcus constellatus, Streptococcus pneumonia*, *Streptococcus mitis*, and *Neisseria meningitidis* can cause invasive diseases such as pyogenic liver abscesses (Kokayi, 2021; Samra et al., 2021), meningitis (Chang et al., 2002; Ostergaard et al., 2005; Kutlu et al., 2008), and pneumonia (Shinzato and Saito, 1995; Catterall, 1999; Glikman et al., 2006). Co-infection can increase the susceptibility of COVID-19 patients to these invasive diseases by decreasing lymphocyte and host immune function and increasing resistance to antibacterial therapy. Thus, the complex nature of pathogen co-infections and host interactions is associated with various clinical symptoms and increases the risk of shock, respiratory failure, prolonged stay in the intensive care unit, and mortality.

However, with an increasing number of patients recovering, it is urgent to focus on the physical status of recovered patients, especially the respiratory immune system. Understanding the changes in the immune status and microbiome of the respiratory tract after recovery is essential for improving diagnosis and treatment. Although analysis of the KEGG pathways associated with the differentially expressed genes between the infected and recovered groups did not show any significant enrichment, some upregulated genes in the infected group compared with the recovered group were involved in immune-related functions (**Figure 1B**), including *IL17RD* (interleukin 17 receptor D), *CD74* (CD74 molecule), *NTRK2* (neurotrophic receptor tyrosine kinase 2), *NKD1* (NKD inhibitor of WNT signaling pathway 1), *CD180* (CD180 molecule), and *TNFSF15* (TNF superfamily member 15). Previous studies have shown that IL17RD, which is similar to Fgf genes (SEF), acts as a signaling hub that negatively regulates mitogenic signaling pathways, such as the ERK1/2 MAP kinase pathway and innate immune signaling (Girondel et al., 2021). Furthermore, studies have demonstrated that IL-17 can activate NF-κB transcription factors in many cell types (Shabgah et al., 2014). IL-17 in humans is associated with the pathology of numerous autoimmune and inflammatory conditions, such as rheumatoid arthritis (RA) and multiple sclerosis (MS) (Tesmer, 2008). Additionally, transcriptomic analysis of the innate immune signatures of a SARS-CoV-2 protein subunit vaccine ZF2001 and an mRNA vaccine RRV revealed that ZF001 induced MHC class II-related genes, including CD74 and H2-Aa, more expeditiously than did RRV (Wang et al., 2022). A summary data-based Mendelian randomization (SMR) analysis and a transcriptome-wide association study (TWAS) were performed for the severe COVID-19 dataset, and seven novel genes for severe COVID-19 were identified, including CCR5, CCR5AS, IL10RB, TAC4, RMI1, and TNFSF15 (Rao et al., 2021). Thus, we speculate that IL-17RD, CD74, and TNFSF15 might serve as disease biomarkers for COVID-19.

A previous study demonstrated that host transcriptome profiles helped build a host transcriptional classifier (*NFAT-5*, *ZC3H11A*, and *PRRC2C*) for diagnosing lower respiratory tract infections with an AUC of 0.88 (95% CI, 0.75-1.00) (Langelier et al., 2018). These host genes are involved in immune system regulation. The sample size of this study was small, but it still makes a critical contribution to monitoring COVID-19 patients receiving treatment. The MDA algorithm was used to further validate the classifier associated with COVID-19 recovery, which contributed significantly to prediction performance. The predictive model of 10 genes (nine known genes and one uncharacterized gene) revealed remarkable discriminating power and exhibited potential for indicating disease treatment. For example, two-pore domain potassium channels encoded by *KCNK12* enable background leakage of K^+,^ which is important for baseline cellular activity at rest, including membrane potential, calcium homeostasis, and cell volume regulation (Williams et al., 2013). Not only can the gene copy number of *KCNK12* allow it to serve as a molecular biomarker for the early detection of diseases (acute lymphoblastic leukemia (Han and Kang, 2009), non-Hodgkin’s lymphoma (Han and Kang, 2009), tubular breast carcinoma (Riener et al., 2008), synovial sarcoma (Heidecker et al., 2010), and malignant peripheral nerve sheath tumors (Nakagawa et al., 2006)) but the expression of these two-pore domain potassium channels also holds promise as an index for therapeutic targets. To the best of our knowledge, this is the first study to identify *KCNK12* as a host classifier for COVID-19. In contrast, severe acute respiratory syndrome-related coronaviruses, *Agrobacterium tumefaciens, Klebsiella michiganensis, Acinetobacter pittii, Bacillus* sp. FJAT.14266, *Brevundimonas naejangsanensis, Pseudopropionibacterium propionicum, Priestia megaterium, Dialister pneumosintes, Veillonella rodentium*, and *Pseudomonas protegens* were selected as candidate microbial markers to trace patient recovery. Ren et al. indicated that *Porphyromonas*, *Haemophilus*, and *Family_XIII_incertae_sedis* carried high levels of oral microbial markers during recovery from COVID-19 (Ren et al., 2021). These similar results indicate that interventions informed by these bacteria may impact patient outcomes. This molecular detection strategy is highly accurate and efficacious. In the future, we might be able to assist physicians in monitoring COVID-19 and predicting disease progression *via* next generation sequencing (NGS).

## Conclusion

Although the sample size is small and the sample source is narrow, this study contributes significantly to the exploration of the microbiome which is closely related to COVID-19. We identified compositional and functional alterations in the COVID-19-associated microbiome, identified specific biomarkers in humans, explained the mechanism of co-infection, and developed a diagnostic strategy. The microbiome also plays a vital role in COVID-19 recovery. Therefore, we developed gene-based and microbial-assisted methods to guide antibiotic treatment and monitor patient treatment status. With the progress of NGS and further study of the possible mechanisms by which the microbiome affects diseases, the use of gene-based and microbial-assisted diagnosis, treatment, and the prognosis is promising for COVID-19.

## Data availability statement

The data presented in the study are deposited in the National Genomics Data Center (NGDC) repository, accession number PRJCA012894, https://ngdc.cncb.ac.cn/search/?dbId=bioproject&q=PRJCA012894&page=1.

## Ethics statement

The studies involving human participants were reviewed and approved by Beijing Ditan Hospital. The patients/participants provided their written informed consent to participate in this study.

## Author contributions

All the authors contributed to the research significantly. RJ and YW conceived and designed the analysis; XX and DW collected the samples; SW and JG tested the samples. HM performed the analysis and CR, YG, HL assist with the bioinformatic analysis; HM wrote the first draft, SW revised the manuscript. All the authors provided critical advice to the revision of the manuscript.

## Funding

This work was supported by Beijing Municipal Natural Science Foundation (grant No.2022-2-014) and Beijing high-level public health technical personnel construction project (grant No.M21003).

## Conflict of interest

Author CR, YG and HL was employed by the company Guangzhou Vision Medicals Co.LTD.

The remaining authors declare that the research was conducted in the absence of any commercial or financial relationships that could be construed as a potential conflict of interest.

## Publisher’s note

All claims expressed in this article are solely those of the authors and do not necessarily represent those of their affiliated organizations, or those of the publisher, the editors and the reviewers. Any product that may be evaluated in this article, or claim that may be made by its manufacturer, is not guaranteed or endorsed by the publisher.

## Supplementary material

Supplementary Figure 1 The differences in immune cell abundance across stages for Neutrophils **(A)**, and Macrophages M0 **(B)**, M1 **(C)**, and M2 **(D)**. The *p values* were calculated using DESeq2.

Supplementary Figure 2 The performance of the random forest classifier for the Infected vs. Other **(A, B)** and Other vs. Recovered **(C, D)** groups with differential genes **(A, C)** and differential microbial taxa **(B, D)** as features. Mean ROC curves are shown with ± 1 standard deviation in gray shading. AUC, area under the curve; ROC, receiver operating characteristic; std. dev., standard deviation.

Supplementary Table 1 Mapping information of sequencing data.
