# Supplementary figures and images for: Respiratory immune status and microbiome in recovered COVID-19 patients revealed by metatranscriptomic analyses
Source: Front Cell Infect Microbiol. 2022 Nov 22;12:1011672. doi: 10.3389/fcimb.2022.1011672 (PMC9724627; doi:10.3389/fcimb.2022.1011672)

**A**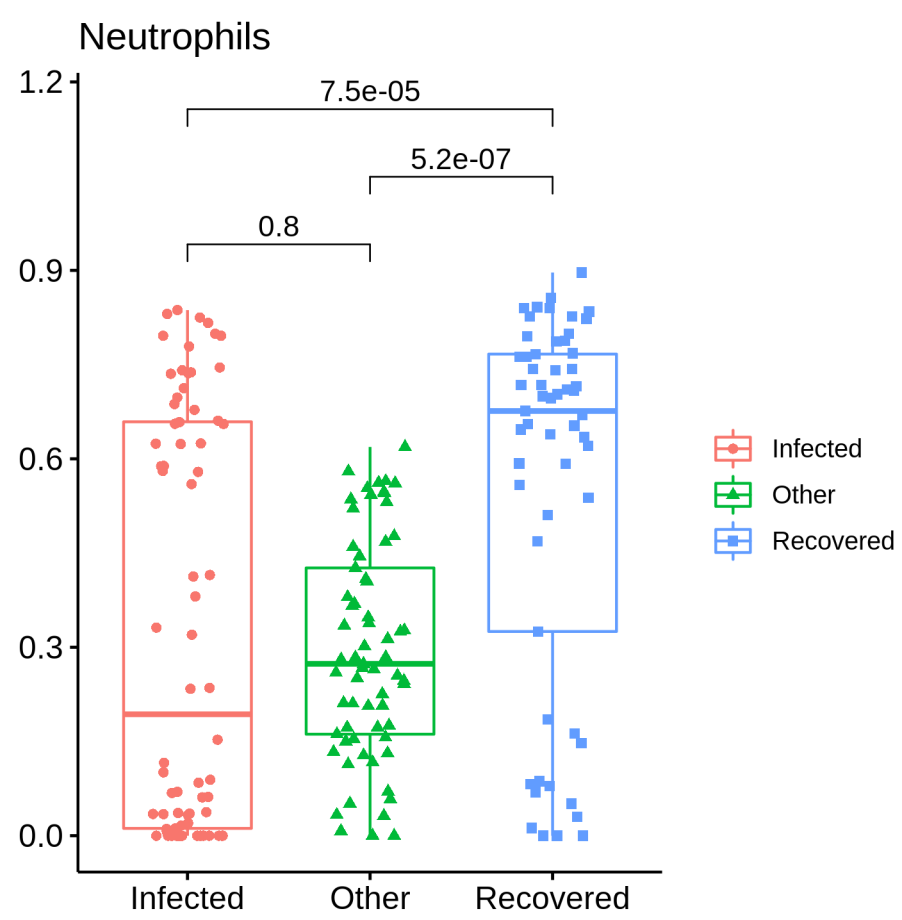**B**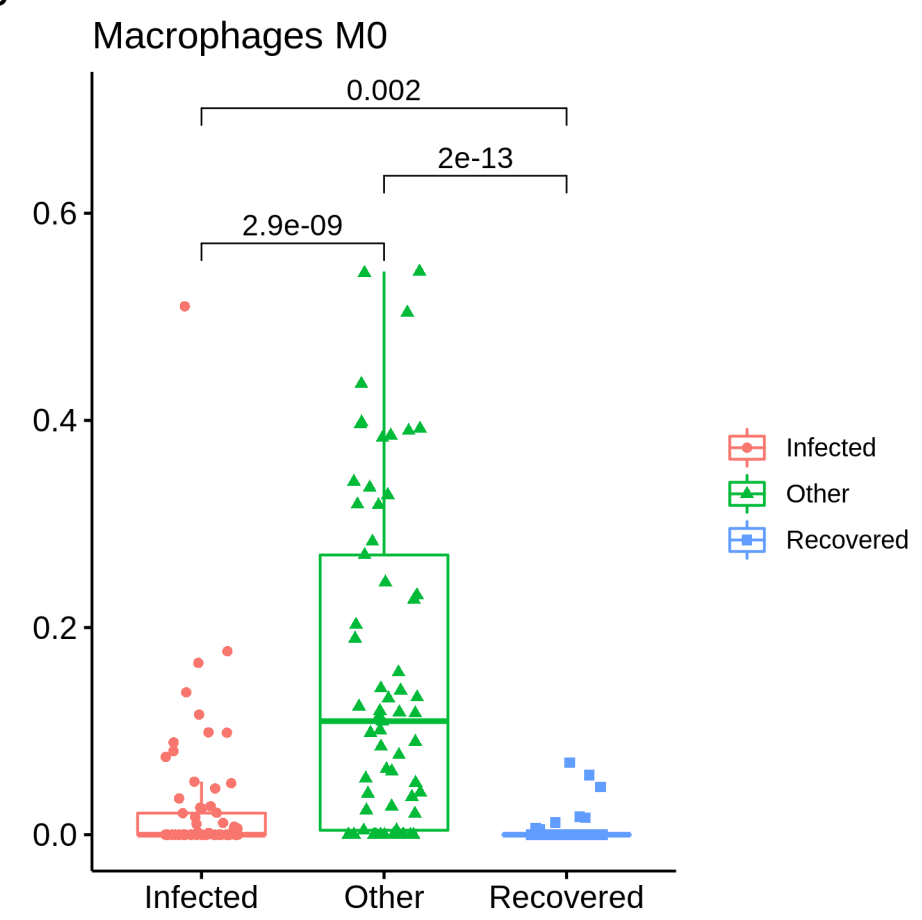**C**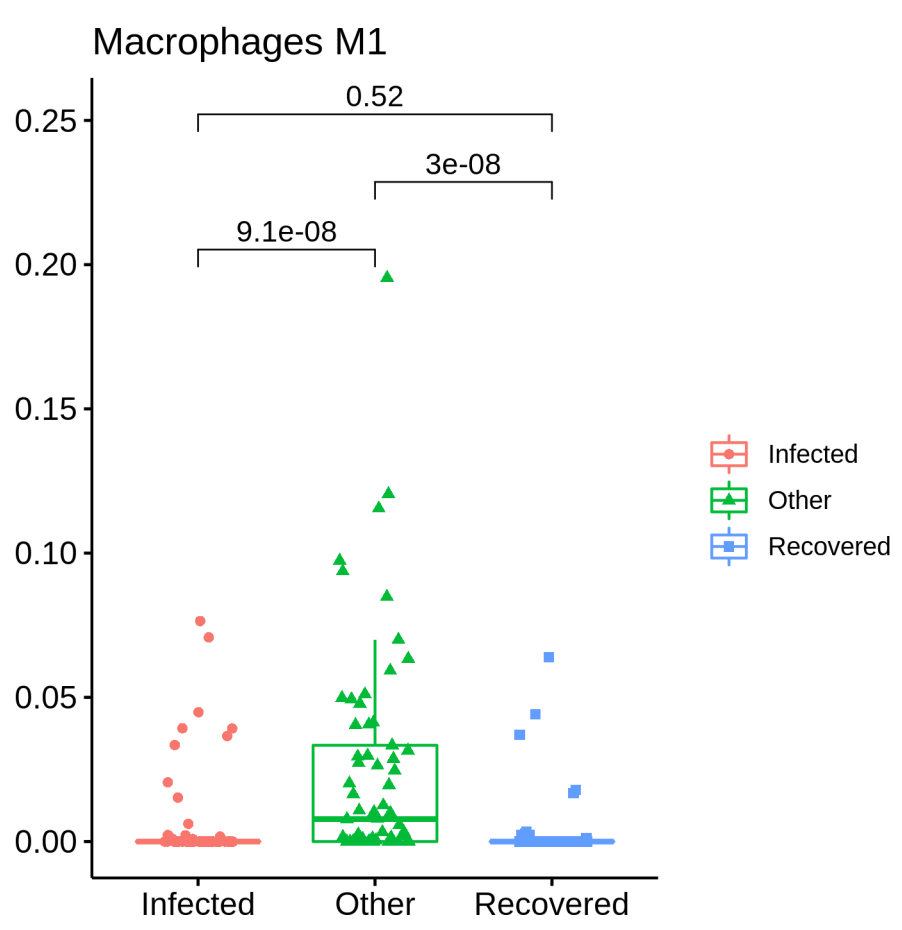**D**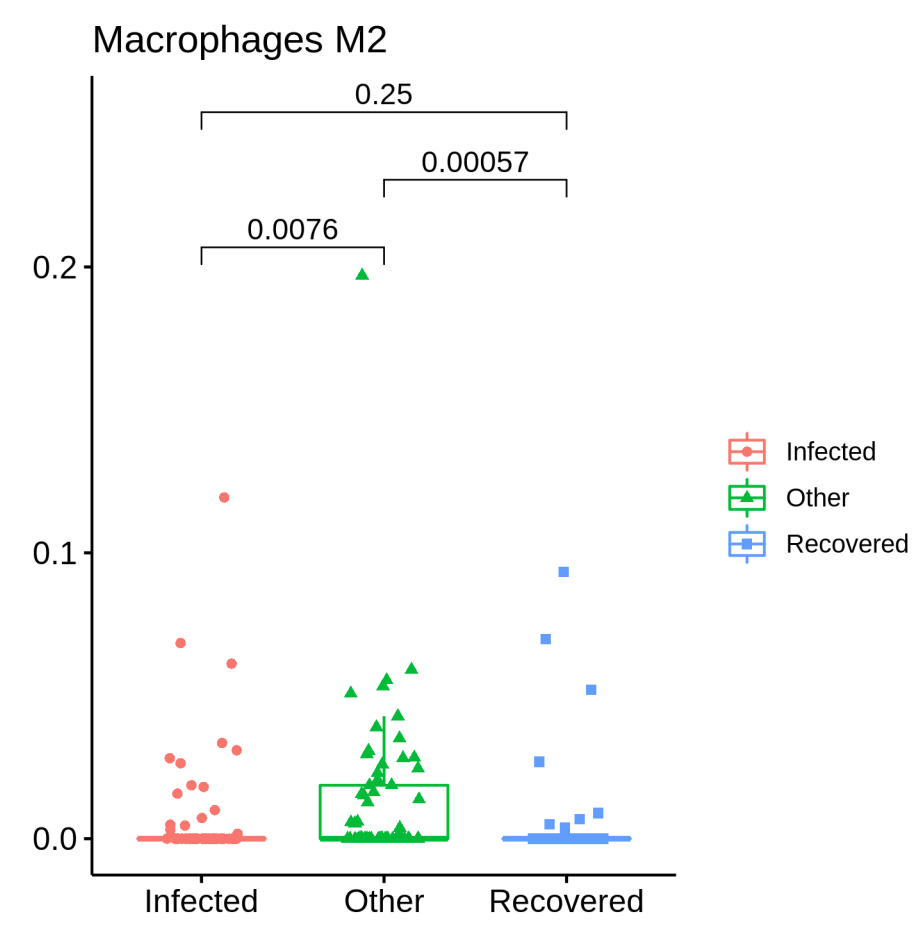

Supplement: Supplementary Figure 1 — The differences in immune cell abundance across stages for Neutrophils (A), and Macrophages M0 (B), M1 (C), and M2 (D). The p values were calculated using DESeq2. [file DataSheet_1.pdf]

**A**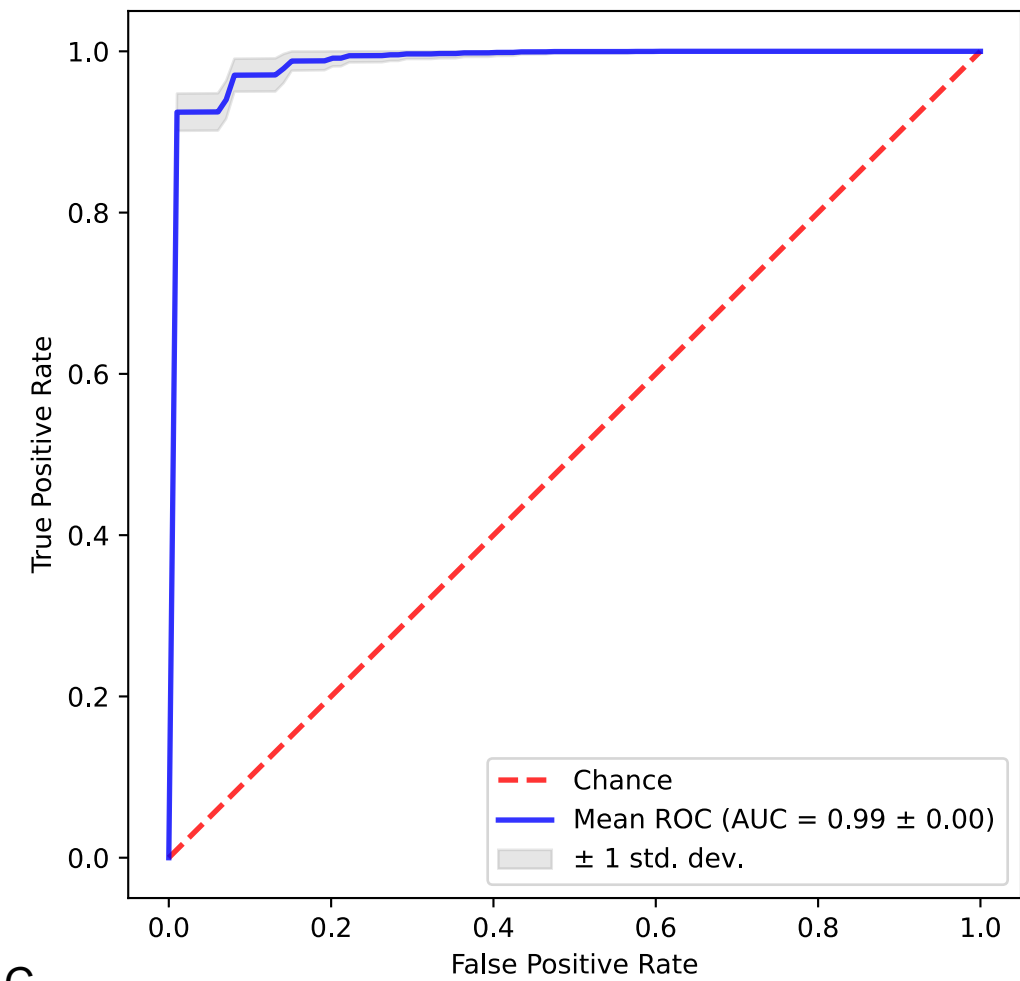**B**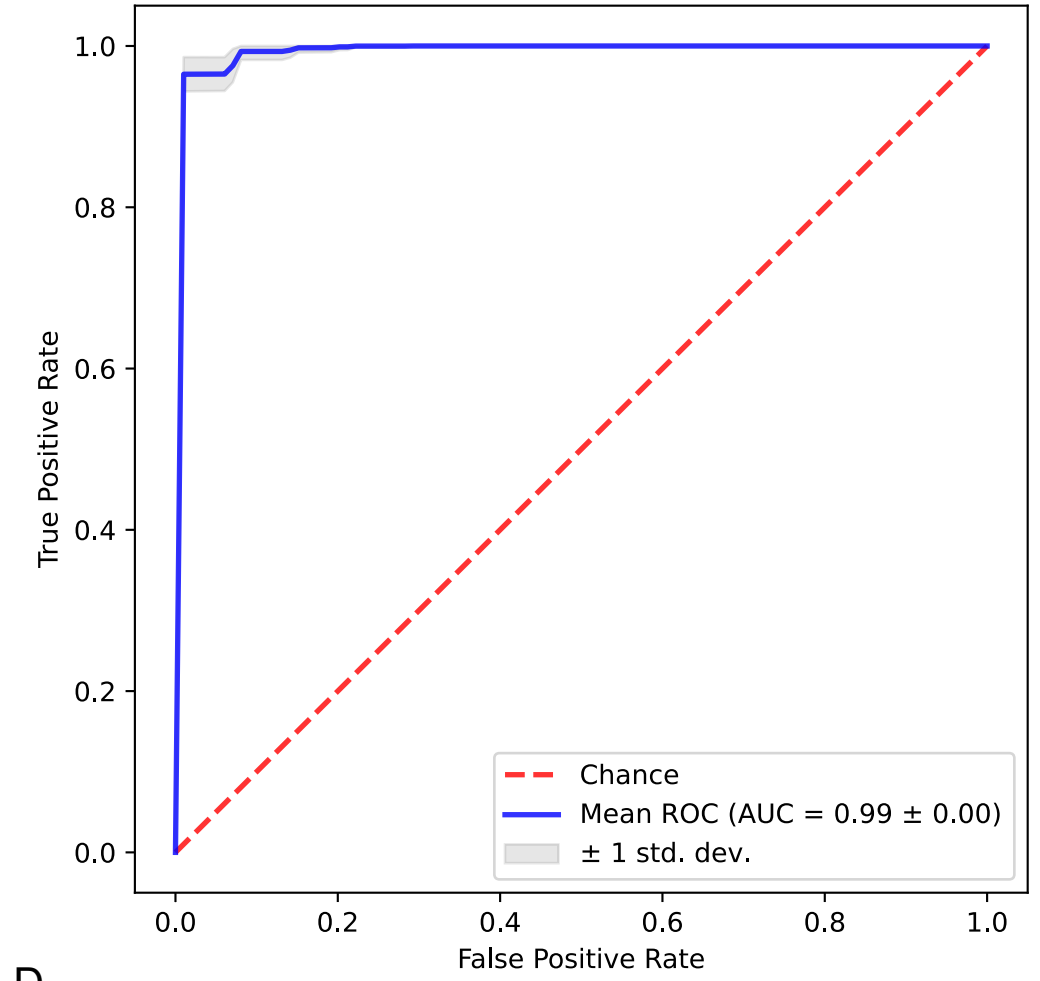**C**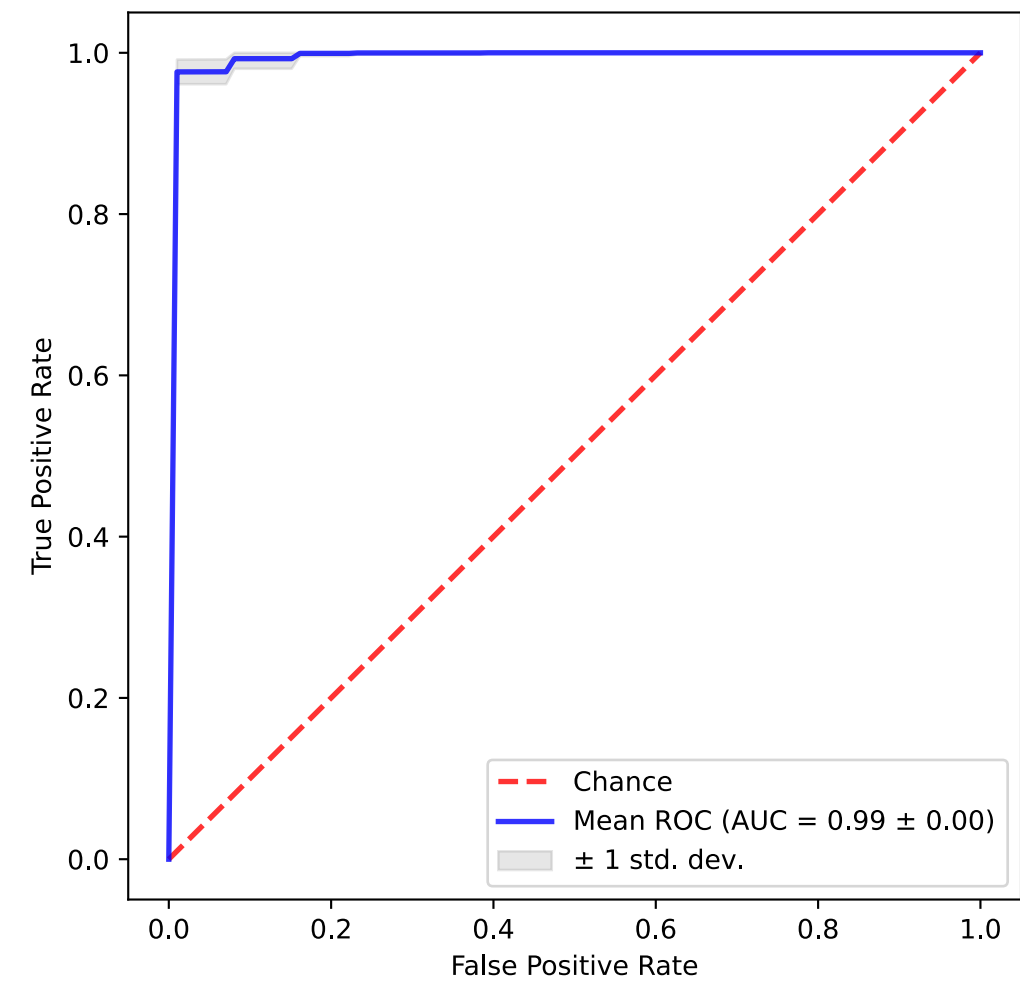**D**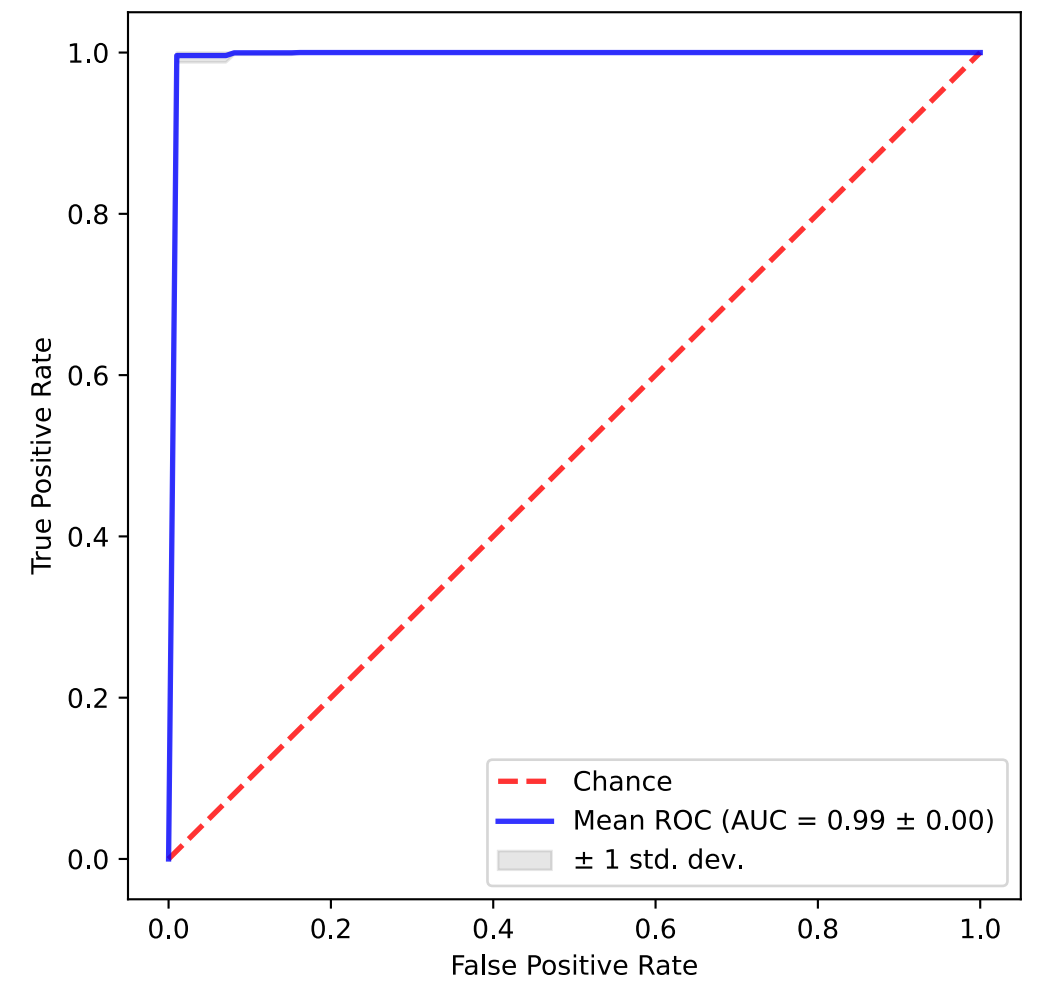

Supplement: Supplementary Figure 2 — The performance of the random forest classifier for the Infected vs. Other (A, B) and Other vs. Recovered (C, D) groups with differential genes (A, C) and differential microbial taxa (B, D) as features. Mean ROC curves are shown with ± 1 standard deviation in gray shading. AUC, area under the curve; ROC, receiver operating characteristic; std. dev., standard deviation. [file DataSheet_2.pdf]
